# The Impact of Grammar on Mentalizing: A Training Study Including Children With Autism Spectrum Disorder and Developmental Language Disorder

**DOI:** 10.3389/fpsyg.2019.02478

**Published:** 2019-11-19

**Authors:** Stephanie Durrleman, Morgane Burnel, Jill Gibson De Villiers, Evelyne Thommen, Rachel Yan, Hélène Delage

**Affiliations:** ^1^Department of Psycholinguistics, University of Geneva, Geneva, Switzerland; ^2^Department of Linguistics, University of Geneva, Geneva, Switzerland; ^3^Laboratoire de Psychologie et Neurocognition, Department of Psychology, Université Grenoble Alpes, Saint-Martin-d’Hères, France; ^4^Department of Psychology, Smith College, Northampton, MA, United States; ^5^EESP, University of Applied Sciences and Arts, Western Switzerland, Lausanne, Switzerland

**Keywords:** Autism Spectrum Disorder (ASD), Developmental Language Disorder (DLD), Theory of Mind (ToM), sentential complements, training program

## Abstract

Training on complements in English, German, and Mandarin has been reported to trigger improvements on both complements and Theory of Mind (ToM), with typically developing (TD) pre-schoolers on the verge of developing these skills (Hale and Tager-Flusberg, 2003; Lohmann and Tomasello, 2003; Shuliang et al., 2014). In the current study, we build on the idea that increasing mastery of complementation holds the promise of enhancing ToM, and seek (i) to replicate the positive effects observed in previous work for this effect in French-speaking TD children, and (ii) to pilot extending this to clinical children, more specifically those with Autism Spectrum Disorder (ASD) and Developmental Language Disorder (DLD), through exploring whether improvement in the latter, clinical groups follows that of the TD group. Sixty children with ToM difficulties, 16 with ASD (aged 5;6–11;8), 20 with DLD (aged 4;8–9;0) and 24 typically developing children aged (2;9–5;3 years), participated in a 4-week training program. Half received training targeting sentential complements and half received a control training targeting lexical skills. Complementation training, but not lexical training, led to a significant direct increase in complements, and also had the indirect effect of significantly boosting belief reasoning. TD and clinical groups followed the same patterns of performance. These results confirm previous findings in other languages for TD, and further suggest promising new directions for therapeutic programs addressing ToM delays in populations of different aetiologies, namely the incorporation of a motivating training on complementation.

## Introduction

The ability to grasp that people’s mental representations of the world may or may not correspond to reality is an important milestone in the development of ‘theory of mind’ (ToM) (Dennett, 1978; Premack and Woodruff, 1978; Flavell, 1999). Typically developing (TD) preschool children struggle at tasks requiring them to predict another’s actions based on their false belief (FB) (Wellman et al., 2001). Such tasks usually involve a protagonist whose mistaken belief about an object has arisen because (1) the object was displaced (Change of Location Task, Wimmer and Perner, 1983; Baron-Cohen et al., 1985) or (2) the object has the appearance that it might contain something else than its actual contents (Unexpected Contents, Gopnik and Astington, 1988).

(1) Sally places a ball in a basket, then leaves. While she is absent, Anne arrives and moves the ball from the basket to a box. Sally returns, and the children are asked: (a) Where Sally will look for her marble? (The critical “belief” question), (b) Where it is really? (The “reality” question), (c) Where it was at the beginning? (The “memory” question).
(2) After being presented with a Smarties tube, the children are asked what they think is inside, to which they typically reply: ‘Smarties.’ It is then revealed that in fact there are pencils inside, at which point the child is asked if s/he can remember the contents of the tube, as well as the critical test question: What would another person would think is inside?

Accurate responses during tasks such as those above are only attested around the age of 4–5 years in TD (Wellman et al., 2001; Milligan et al., 2007). It is important to emphasize the conceptual difficulty involved during FB attribution: the child must reconcile the contradiction between what s/he knows and what the other believes. This is a sophisticated step, preceded by simpler mental states attributions. The attribution of diverse desires and beliefs, for instance, does not require the child to reconcile a perspective in contradiction with what they know to be true, and these FB-precursors emerge earlier in development than the attribution of FB, i.e., before age 4 (Wellman and Liu, 2004).

The emergence of successful mentalizing including FB reasoning is important for the development of social cognition on various levels, e.g., fluid conversational skills, conflict resolution, popularity amongst peers, etc. (Astington and Jenkins, 1999; Astington, 2003; Astington and Pelletier, 2005; Astington and Edward, 2010; Mazza et al., 2017; Derksen et al., 2018). In certain clinical populations, such as children with Autism Spectrum Disorder, difficulties with FB reasoning often persist later in development, affecting performance on FB tasks even at a mental age of 9 years (Baron-Cohen et al., 1985). This marked delay has been interpreted to indicate a core mind-reading deficit (Baron-Cohen, 1990), which would explain weaknesses in communicative and social skills characteristic of the autistic condition (Diagnostic and Statistical Manual of Mental Disorders, 5th edition). However, a subset of children with ASD, from 20 to 50% (Baron-Cohen et al., 1985; Prior et al., 1990), systematically succeeds at FB attribution and thus arguably can surmount their fundamental ToM difficulty (Tager-Flusberg and Joseph, 2005). It has been claimed that in order to accomplish this, they apply verbal strategies. Put differently, children on the spectrum could use language to support their reasoning about others’ beliefs, with some studies suggesting that lexical abilities play a role in ToM (e.g., Happé, 1995), and others pointing rather to the importance of grammatical skills (Fisher et al., 2005; Milligan et al., 2007). The linguistic determinism approach (de Villiers, 2007) maintains that a specific grammatical structure is most crucially solicited during mentalizing, namely complement clauses such as (3), where a proposition is embedded under a verb of mental-state (e.g., *think*, *believe*) or communication (e.g., *say, mention*):

(3) That doll thinks/ believes / says/ mentions that [her ball is in the basket / some Smarties are in this tube]

This linguistic tool would serve to efficiently represent subjective truths because the content of the complement (in brackets) has an independent truth-value, and consequently can be false while the entire sentence remains true. These semantic and syntactic properties render complements ideal tools for grasping propositional attitudes and thus efficiently representing subjective truths (Perner, 1988; de Villiers et al., 2014), albeit with some cross-linguistic variation (Perner et al., 2003; Cheung et al., 2004; Tardif et al., 2007).

In support of the view that complementation assists complex ToM reasoning, authors have reported links between mastery of this structure and success at FB in young TD children (de Villiers, 2000; de Villiers and de Villiers, 2000) as well as in children with ASD (Tager-Flusberg, 2000; Tager-Flusberg and Joseph, 2005; Lind and Bowler, 2009) and language-delayed deaf children (Schick et al., 2007). Interestingly, these links are also found when the complements do not occur with mental state verbs but rather with verbs of communication, which themselves do not refer explicitly to mental states (de Villiers and Pyers, 2002; Durrleman and Franck, 2015). Indeed the latter verbs, being less abstract than mental state verbs, have even been argued to be most crucial for ToM success in children with ASD (Tager-Flusberg and Joseph, 2005). Knowledge of sentential complements, rather than of mental state lexicon, would therefore allow children to bootstrap their meta-representational grasp of beliefs.

If language skills, in particular with complementation, serve for belief reasoning, then populations with language difficulties that include complementation would also be expected to struggle with this aspect of ToM. This seems to be the case for children with Developmental Language Disorder (DLD). Children with this condition display primary difficulties in formal language (Leonard, 2014) including complementation (Tuller et al., 2012; Steel et al., 2016) and are also reportedly delayed in ToM, even if these delays appear to be more subtle than those attested in ASD (Holmes, 2002; Tucker, 2004; Andrés-Roqueta et al., 2013). Moreover, mastery of complements by children with DLD also relates to their success at ToM as measured by false-belief tasks (de Villiers et al., 2003; Miller, 2004). Interestingly, the verbal demands of the ToM tests administered in the studies conducted with this population may impact their performance (Miller, 2001), but these alone do not suffice to clearly explain their ToM performance, as even tasks that rely minimally on language pose problems, suggesting that the difficulty is at the level of ToM reasoning (Nilsson and de López, 2016). In favor of the view that language influences ToM reasoning and not only verbal ToM task performance, relations between complements and low verbal ToM tasks have been reported for both DLD (Durrleman et al., 2017a) and ASD (Durrleman et al., 2016a). Taking as a point of departure that language is not only fundamentally related to mentalizing, but also influences its development rather than vice versa as revealed by longitudinal studies (TD: Astington and Jenkins, 1999; ASD: Tager-Flusberg and Joseph, 2005), researchers have aimed to trigger ToM via the training of complements in preschool TD children. Results have revealed that this training is indeed effective at boosting ToM, even when training involved complements of verbs of communication alone (Hale and Tager-Flusberg, 2003; Shuliang et al., 2014) and when deceptive scenarios (i.e., involving appearance-reality dissociations) were not included to train complements (Lohmann and Tomasello, 2003; Shuliang et al., 2014), although capitalizing on both complements and deceptive scenarios together appears to be especially useful for consolidating ToM. Still, none of these studies on complementation training included participants delayed for either language or ToM, and instead focussed on children on the cusp of developing these skills anyway. It thus remains to be determined whether populations where ToM and/or language is affected would show similar boosts in belief reasoning to that already observed in TD children due to complementation training. The current work is thus concerned with elucidating whether training sentential complements can be beneficial for the remediation of belief reasoning in children with ASD and those with DLD, along the lines of TD. It is also an open question whether enhancing complementation can also be useful for other aspects of ToM beyond false belief reasoning, such as grasping diversity of desires.

In the current study, we build on the idea that increasing mastery of complementation holds the promise of enhancing ToM, and seek (i) to replicate the positive effects observed in work on other languages for this effect in French-speaking TD children, and (ii) to pilot extending this to clinical children, more specifically those with ASD and DLD, through exploring whether improvement in the latter, clinical groups follows that of the TD group. If this proves to be the case, our results would suggest a novel, evidence-based, clinical intervention, addressing both language and ToM in these populations.

We explore several other questions as well with our rich data set. We verify that complementation training is more effective for complements and ToM than a more general, lexical training. We test whether the effects of complement training are particular to false beliefs assessed verbally, or encompass low-verbal false beliefs too. We ask whether the contribution of complementation is specific to false belief reasoning, or whether it can be observed to assist other, earlier-mastered aspects of ToM, like diverse desires and true beliefs. Importantly for clinical purposes, we ask whether the hypothesized ToM gains persist through time by retesting after a delay. Finally, we ask whether the control group, who received lexical training, differentially improved to the target, complementation training group, on the lexical tasks.

In addition to individual analyses on the outcome measures, we undertake a Structural Equation Model (SEM) to look more closely at the pathways of change, for example, asking whether the success of complement training depended on other abilities, such as non-verbal reasoning or language skills at outset. SEM allows several advantages over simple regressions or ANOVAS, especially when variables are highly intercorrelated, as they are in this study. It models the relationships among multiple independent and dependent variables simultaneously, unlike linear regression, which can only analyze one layer of linkages at a time. Because SEM can test multiple pathways, it allows the investigation of both direct and indirect effects in one hypothesized model (Gefen et al., 2000). This is important in determining the particular role that the complement training plays in advancing false belief understanding.

## Materials and Methods

### Participants

All of the participants in this study were native French-speakers, recruited in Geneva and Lausanne, Switzerland and Paris, France. The project received approval from the Ethics Committee of the Faculty of Psychology and Educational Sciences of the University of Geneva as well as from the Geneva Cantonal Ethics Commission, and was also declared at ‘La Commission Nationale de l’Informatique et des Libertés (CNIL)’ in France. Children’s parents all provided written, informed consent for their child to participate.

Sixty children participated in the study: 16 children with ASD aged 5;6–11;8 (*M* = 8;3), 20 children with DLD aged 4;8–9;0 (*M* = 6;9) and 24 TD children aged 2;9–5;3 years (*M* = 4;3). Differences in age were due to the fact that difficulties on ToM have been attested at different phases of development in these three populations. Matching was done on the groups’ linguistic and cognitive characteristics as explained below. Children with ASD were recruited from specialized schools, those with DLD from speech-language centers which they attended after school, and TD children from kindergartens and day-care facilities. We targeted children of the age range when complements and ToM are reportedly not yet mastered, hence for TD children this meant choosing children between the ages of 3 and 6 years (Wellman et al., 2001), for children with DLD the upper cut-off was age 9 (Andrés-Roqueta et al., 2013; Nilsson and de López, 2016) and for children with ASD this cut-off was extended to 12 years (Baron-Cohen et al., 1985; Yirmiya et al., 1998). Then, for these children to be included in the study, they had to meet several criteria: (i) TD children had to have no history of language impairment and needed to be included in normal classrooms without support. (ii) In contrast, clinical groups had to have been previously given the relevant diagnosis by a qualified professional. More specifically, children with DLD needed to have obtained language scores of at least 2 SDs below age-specific norms according to standardized tests used by speech and language pathologists in Switzerland and France (CIM 10; De La Santé, 1993), while children with ASD had to have met the criteria for this condition according to the DSM-IV-TR (American Psychiatric Association [APA], 2000), the Autism Diagnostic Observation Schedule, ADOS (Lord et al., 2003) and/or the ADI-R (Rutter et al., 2003). (iii) Scores on pre-(training)-tests assessing ToM and complements also had to leave enough of a margin for progress to be achieved, thus only children performing equivalent to or below 70% were included (equivalent to a maximum of 8 successful items out of 12 on FB and 4 successful items out of 6 on false complements). (iv) In addition, parents had to report that their child’s language comprehension was of the level to understand simple subject-verb-object sentences, which was subsequently confirmed by experimenters upon the first meeting during language tasks (Exalang et al., 2006), such that leading them up to complex sentences in a relatively short space of time could be feasible. (v) Finally, only children who could attend to pre-tests could proceed to training.^1^

Within each population, one half was assigned to the target-training program involving the teaching of sentential complements, while the other half was assigned to an alternative training program focussing on lexical enrichment. The latter group allowed us to confirm that any effects arising with complementation training were not due to general linguistic stimulation. This preliminary study involved small groups of participants for each population of children. Because our hypotheses were identical for all of these populations, we analyzed their data grouped together and focused on the type of training, and then conducted analyses to see whether the overall results were driven by any subgroup/specific population(s), i.e., whether progress in the TD and clinical groups were similar.

Amongst the target-training group, there were 21 boys and 9 girls and amongst the control-training group there were 20 boys and 10 girls. The two groups of 30 were matched on a variety of global cognitive and linguistic standardized measures (all *t* < 1, see Table 1 for precise *p*-values), namely non-verbal reasoning (Raven et al., 1998), as well as general morphosyntax and lexicon (Exalang 3–6; Helloin and Thibault, 2006). For more specific measures, we created tests assessing: (i) complementation understanding (based on de Villiers and Pyers, 2002) and (ii) ToM abilities. The latter included a verbal measure of false-belief (based on Baron-Cohen et al., 1985), a minimally (low-)verbal measure of false-belief (based on Woolfe et al., 2002) as well as a test assessing skills emerging just before false-belief reasoning (FB precursors), namely diverse desires and diverse beliefs (based on Burnel et al., 2017). We refer to the latter as low-verbal ToM and FB precursors. Table 1 presents the descriptive measures of the children included in each of the training groups. Details on the descriptive characteristics of the cognitive groups (TD, ASD and DLD) are reported in Table 2. While these groups differ for age [*F*(2,52) = 37.57, *p* < 0.001], for reasons explained above, they do not differ on standardized measured of non-verbal reasoning (*p* = 0.09), and morphosyntax (*p* = 0.23) or lexicon (*p* = 0.27).

**TABLE 1:** Means (standard deviations) on paired variables at the moment of pre-test for the two groups (syntactic training, lexical training) and the three populations of children (TD, Typically Developing; ASD, Autism Spectrum Disorder; DLD, Developmental Language Disorder).

	**Syntactic training**			**Lexical training**			***T*-tests results**
	***n* = 30**			***n* = 30**			
	**TD**	**ASD**	**DLD**	**TD**	**ASD**	**DLD**	
	***n* = 12**	***n* = 8**	***n* = 10**	***n* = 12**	***n* = 8**	***n* = 10**	
Chronological Age	5.92 (1.97)			6.57 (2.36)			*t*(58) = 1.16, *p* = 0.25
	4.29	7.52	6.58	4.35	9.04	7.25	
Raven	15.53 (5.26)			15.87 (4.90)			*t*(58) = 0.25, *p* = 0.80
	12.92	16.25	18.10	14.50	15.63	17.70	
Global morphosyntax	10.62 (2.54)			10.97 (1.94)			*t*(56) = 0.58, *p* = 0.56
	10.00	10.57	11.40	10.58	10.29	11.90	
Global lexicon	34.2 (2.8)			32.8 (6.4)			*t*(58) = 1.13, *p* = 0.27
	33.8	34.4	34.5	33.7	30.4	33.6	
False Complements/6	1.47 (1.50)			1.57 (1.48)			*t*(58) = −0.26, *p* = 0.79
	0.92	1.50	2.10	1.17	2.13	1.60	
Verbal FB/6	0.63 (0.76)			0.93 (1.17)			*t*(58) = −1.18, *p* = 0.25
	0.50	0.75	0.70	0.90	1.50	0.70	
Low-verbal FB/6	2.60 (1.90)			2.73 (2.18)			*t*(58) = −0.25, *p* = 0.80
	2.58	3.13	2.20	2.50	4.13	1.90	
FB Precursors	4.77 (1.48)			4.69 (1.65)			*t*(57) = 0.19, *p* = 0.85
	4.25	5.38	4.90	3.50	5.88	5.22	

**TABLE 2:** Descriptive characteristics of participants.

	**TD (*n* = 24)**	**ASD (*n* = 16)**	**DLD (*n* = 20)**
Chronological Age	4.32 (0.67)	8.28 (2.02)	6.92 (1.53)
Raven	13.71 (4.72)	15.94 (15.94)	17.90 (4.54)
Global morphosyntax	10.29 (2.29)	10.43 (2.06)	11.65 (2.16)
Global lexicon	33.75 (3.17)	32.38 (8.31)	34.05 (2.72)

#### Material and Procedure

**FIGURE 1:**
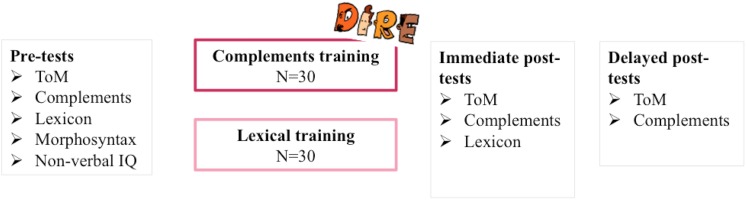
Experimental design.

Pre-tests assessed a series of relevant measures, namely ToM (via verbal and low-verbal FB tasks as well as a mini-test of FB precursors), complements, lexicon, morphosyntax and non-verbal reasoning (see below for more details on these measures). One to two weeks after being tested for the first time, participants were randomly assigned to one of the two training programs, i.e., either that of complements or lexicon. Each program lasted 4–6 weeks. One to two weeks after training ceased, immediate post-(training)-tests were administered to determine potential gains on abilities targeted by the programs, namely complements, ToM and lexicon. Again 4–6 weeks went by, this time without training, and another set of tests was administered, i.e., ‘follow-up’ or ‘delayed’ tests. These ‘follow-up’ tests were only conducted with children who had made progress of at least 10% between pre-tests and immediate post-tests in order to determine whether gains on complements and ToM could be maintained. Figure 1 outlines the overall experimental design of the study.

### Pre- and Post-tests

All tests, both pre and post, were conducted on laptop computers, to contrast with the materials used for the training itself, which was administered via iPads. This distinction between testing and training modalities allowed us to ascertain that any gains between pre and post-tests could not simply be attributed to increased familiarity with the material used during tests. Tests assessing ToM and sentential complements were specifically designed for the study, but followed the same basic format of tests which have been used in various previous studies, as explained in more details below. Administering ToM tests on a screen as opposed to having them administered by an experimenter held the promise of being the least penalizing option in particular for children with ASD (Chevallier et al., 2014).

Each test of our target variables (i.e., complements and ToM) contained 36 items, which were all animated scenarios. Eighteen of these items involved FB, i.e., they corresponded to the test condition, while the other items involved true beliefs (TB). TB items cannot be taken as unambiguous ToM measures, as accurate responses coincide with reality responses (Dennett, 1978), however, they allowed varying the material, so that children had to adjust their predictions depending on the changing epistemic state of the agent (Forgeot d’Arc and Ramus, 2011). We created three sets of tests, meaning that children who participated in the entire study saw a total of 108 different items (54 FB items and 54 TB items) over the course of three testing sessions, and never saw the same item twice. The order of the items which made up each test was randomized and counterbalanced across participants.

#### Theory of Mind Tests

ToM was evaluated via a total of 12 FB items. These were interspersed with 12 TB items. Of the 12 FB items, 6 formed a *verbal ToM* task and 6 others a *low*-*verbal* one (again 6 true and 6 false beliefs). For each task, the child’s response always implied selecting one element amongst three, two involved in the scenario presented (corresponding to a true vs. a false belief), while the third was unrelated to the story.

**FIGURE 2:**
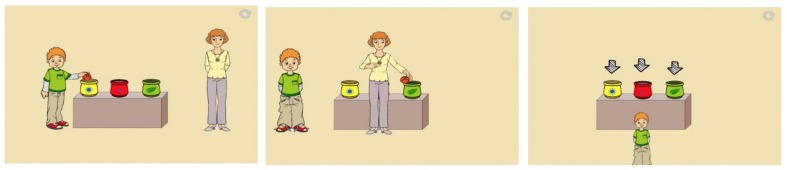
Illustration of the verbal ToM task.

The *verbal ToM* task was directly inspired by the Sally-Anne Task (Baron-Cohen et al., 1985). As explained above, in the FB scenarios of this task, the child is confronted with an object being moved from location 1 to location 2 in the main protagonist’s ignorance and has to capitalize on this protagonist’s false belief to predict that s/he will look for the desired object in location 1 (where it is no longer present). For example, one of the proposed scenes in our assessment was: “This is Bob. This is the mother. Bob has a yellow pot. The mother has a green pot. Bob has a ball, he puts the ball in his yellow pot. Bob is going out to play. The mother takes the ball out of the yellow pot and puts it in the green pot. Now Bob comes back, he wants to play with the ball. Where’s Bob going to get his ball?”. The child must then choose from three answers: the initial position (here the basket), the place where the object is actually located (here the box) and the position of the middle representing an object not involved in the story (here a bag). To succeed, children must take into account Bob’s misrepresentation while putting aside their own knowledge of reality. In the true belief, filler scenarios, the displacement occurred in front of Bob (see Figure 2), or while he was absent the object was manipulated and returned to its original position.

**FIGURE 3:**
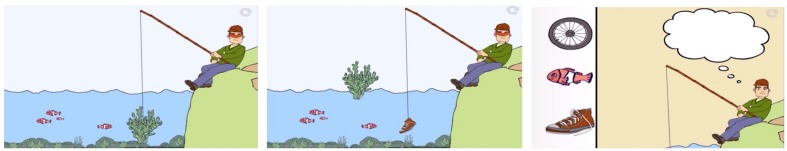
Illustration of the low-verbal ToM task.

The *low-verbal ToM* evaluation was inspired by Woolfe et al. (2002), who claim that the task “minimize(s) verbal task-performance requirements.” This is because the pictures alone are informative enough for the child to both interpret the scene and to respond. As an illustration, images would appear on the screen clearly depicting someone blindfolded who was trying to obtain an object. This information was then also provided verbally in the form of commentary, which was thus not crucial for task success. For example (see Figure 3), in one scene there was a blindfolded man with a fishing rod and seaweed covering the object at the end of his rod and the commentary went: “Look! The man is fishing! He can’t see anything. Let’s see what is behind the seaweed - Click here!” All children understood and clicked, which made the seaweed move aside. In one scenario there was a fish, in another test there was a boot. Then children were then presented with three objects and asked to click on the object the man was thinking about, in this instance selecting between a fish, a boot and a wheel.

#### Complements Test

**FIGURE 4:**
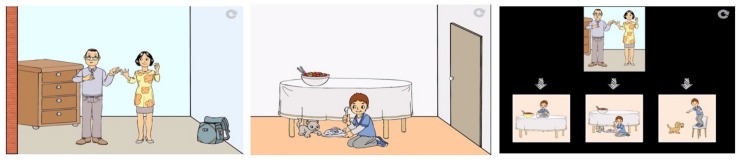
Illustration of the complements test.

The evaluation of sentential complements was inspired by de Villiers and Pyers (2002). The general format involved one protagonist reporting an event to another, after which the actual event was shown. There were a total of 12 items, 6 test FB items and 6 TB items. In the test items, the complement reported an event inaccurately (false complement). The child had to simply recall the content of the erroneous complement uttered in the first scene in order to score a point. An illustration would be: “The mother asks the father what Jean is doing. And the father answers that Jean is eating fish. Look! Jean is giving fish to the cat!” Then, pointing back to the picture of the parents now with three options to select from, the voice said: “Look here: what is Dad saying that Jean is doing?” (see Figure 4). We pointed back to the picture and maintained the present tense in light of observations that past-tense can be difficult for children with DLD (Rice and Wexler, 1996; Bishop, 2013) and ASD (Tager-Flusberg, 1989; Roberts et al., 2004). In the fillers, the report and the event coincided (true complement) such that it sufficed for children just to touch the only event evoked (e.g., the father says that Jean is eating fish and Jean is shown to indeed be eating fish).

#### FB Precursors

If the participant met the inclusionary criteria defined above after the main tests, we administered a mini-test evaluating the skills emerging before the ability to assign false beliefs, namely the understanding that people differ from each other in their desires and beliefs (Wellman and Liu, 2004). There were a total of 6 items seen in each mini-test, 3 diverse desires and 3 diverse beliefs, such that children participating in the three testing sessions saw a total of 18 items assessing FB precursors. As an illustration of an assessment of diverse desires, the child saw an animated story while hearing the following narration: “What do you prefer: a carrot or a biscuit?” The child would then select one (usually the biscuit) and then see a small scene in which another character chooses the opposite, e.g., “Here is Theo. Theo prefers carrots. Theo is hungry. What do you think Theo will eat?”. For an assessment of diverse beliefs, the child heard: “This is Thomas’ book. Sometimes Thomas’ mother puts his book on the table, sometimes Thomas’ mother puts his book on the shelf. This is Thomas. Thomas is looking for his book. Where do you think the book is? On the shelf or on the table?” The child would then click on one, say the shelf, in which case the story would continue: “For Thomas, the book is on the table. Where will Thomas look for his book?”.

### Standardized Tests

#### Non-verbal Reasoning

Raven’s matrices (Raven et al., 1998), were also administered during the pre-test to assess the child’s level of non-verbal reasoning. During this task, the child must complete 36 series of increasing difficulty. Each series is presented with a piece missing, which the child must select amidst six pieces.

#### Language

Finally, we evaluated the child’s language level using a test normed for children aged 3–6 years: EXALANG 3-6 tests (Helloin and Thibault, 2006). We opted for this task to assess receptive lexical skills (via the designation of images) and morphosyntactic (via the morphosyntax subtest) because its general format was very similar to our other tests for ToM and complements, namely they contained simple, computerized animations. Also, as mentioned earlier, belief attribution emerges generally around 4–5 years of age, i.e., along with general language skills corresponding to this age range, thus we reasoned that a language task for this age range would be appropriate for our sample, who were still struggling with FB.

### Training

Training programs either focused specifically on complements (for the target training) or more generally on the lexicon (for the control training). Each involved five types of activities conducted on iPads, two to three times per week for maximum duration of 6 weeks, and a minimum duration of 3 weeks in the event that children already performed at ceiling at this point of the training program.

#### Target Training: Complementation

For the training of complements, we administered a novel iPad application (Durrleman et al., 2016b), called DIRE, which means ‘to say’ in French. This name indicates that the program focuses mainly on the training of complements of verbs of communication (as well as some complements of verbs of perception or desire), thus abstracting away from verbs of mental state such as ‘think’ or ‘believe.’ DIRE also stands for ‘Differentiating Ideas from Reality via Exercises,’ since the purpose of the training offered is to assist children with ToM difficulties to acquire these complements so that they may in turn apply them during ToM reasoning. We opted for iPad training, as such methods have already proven to be effective with clinical populations (Alzrayer et al., 2014). Our training involved five types of activities, various using pictorial representations of speech, as previous work has found that visual cues are effective in remediation programs with ASD (e.g., Wellman et al., 2002; Paynter and Peterson, 2013).

The five activities of DIRE were administered during each training session. The order of appearance of the activities was the same for all children, beginning with activity 1 and ending with activity 5. Each activity addressed a particular aspect of complementation via brief exercises, which are explained in detail in Supplementary Appendix A. The first activity, inspired by Wilson and Fox (2013), dealt with infinitival complements such as: “Sophie sees a baby crying” which are the first kind to be mastered (Bloom et al., 1989; Diessel, 2004). All other activities focused on tensed complements of communication verbs, such as: “The little girl screams that there is a spider in the bathtub” which are the kind specifically hypothesized to support ToM (de Villiers, 2007). Six sessions contained new material, composed of approximately 100 different items. All children were presented with the entire material at least once, and some saw it a second time if the experimenters noticed they were still not excelling after 3 weeks. In this case, material from the beginning would start over.

#### Control Training: Lexicon

The control, lexical training was based on different applications teaching the lexicon, namely *Bitsboard*, Flashcards, *French FEL*, *Apprends-moi les mots* (‘Teach me words”) and *Animaux* (“Animals”). Several themes are covered during the proposed exercises, such as colors, food, means of transport, animals, etc. At each session, we recorded the words learned and thus in subsequent sessions only checked these again before addressing the novel words. This training involved the same sort of demands as the target training, namely image designation, repetition, truth-value judgment, but also carefully steered away from mental state terms.

## Results

### Question 1: Did the Target and Control Training Have Differential Effects?

**FIGURE 5:**
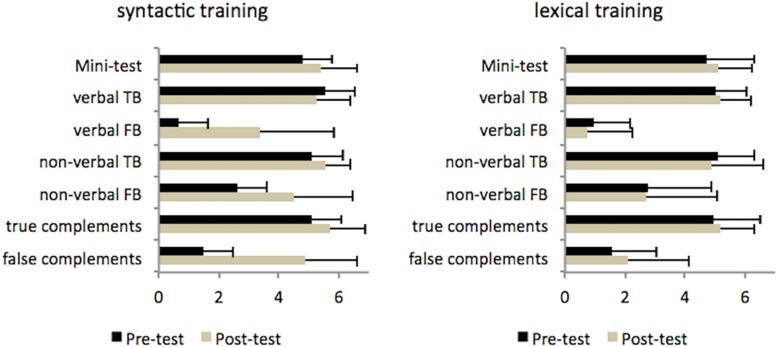
Scores on ToM and complements tasks at pre-test and post-test for the syntactic and lexical groups.

Our first research question concerns whether the trainings had differential effects, namely an improvement from pre-test to post-test specific to the type of intervention (descriptive data for the syntactic and lexical training are provided in Figure 5). Specifically, it is necessary to show that the syntactic training resulted in improvement on false complements, but the lexical training did not. Next, it is necessary to show that the syntactic training has effects on False Belief performance, and that the lexical training did not. Third, it is necessary to show that the training was not restricted to the verbal false belief tasks, but applied equally to the verbal and low-verbal tasks.

Recall that the children were selected to have poor performance on complements and false belief, with the result that the data were non-normally distributed, as the distributions were truncated. To do ANOVAs, we tried using the Box-Cox transformation but homogenous variance and normal error distribution could not be achieved due to this truncation at one end of the distributions. Therefore, on the variables that constituted selection criteria, namely false belief (verbal and low-verbal) and false complements, non-parametric tests were necessary. Wilcoxon signed rank tests were used in the analysis of pre- and post-training effects on the false belief and complement measures for the different training groups. Effects sizes (using r, Rosenthal, 1994) are reported for the non-parametric test and regular Cohen’s *d* (Cohen, 1988) are reported for the parametric test. Using Cohen’s guidelines for r, a large effect is 0.5, a medium effect is 0.3, and a small effect is 0.1 (Cohen, 1988).

In the syntactic training group, Wilcoxon’s signed rank test showed a statistically significant difference between pre-test and post-test on Verbal False Belief (Pre-test: median = 0.50; post-test : median = 4.0; *Z* = −4.07, *p* < 0.001, *r* = 0.74), low-verbal False Belief (Pre-test: median = 2.0; post-test: median = 5.0; Z = −3.42, *p* < 0.001, *r* = 0.63), and False Complements (Pre-test: median = 1.0; post-test: median = 6.0; Z = −4.53, *p* < 0.001, *r* = 0.83) with large effect sizes. However, one-way ANOVAs showed that there was no significant progression between pre-test and post-test on FB precursors [*F*(1,58) = 2.89, *p* = 0.09], Verbal True Belief [*F*(1,58) = 1.08, *p* = 0.30], low-verbal true belief [*F*(1,58) = 2.60, *p* = 0.11], and true complements [*F*(1,58) = 3.20, *p* = 0.08], possibly due to already high scores on these variables (see Supplementary Appendix B).

In the lexical group, one-way ANOVAs showed no significant progression between pre-test and post-test on precursors of FB [*F*(1,56) = 1.46, *p* = 0.23], verbal True Belief [*F*(1,54) = 0.60, *p* = 0.44], low-verbal true belief (*F* < 1), or true complements [*F*(1,55) = 1.36, *p* = 0.25]. Non-parametric tests indicate no significant changes occurred from pre-test to post-test in false complements (*Z* = −1.18, *p* = 0.24, *r* = 0.21), verbal False Belief (*Z* = −1.52, *p* = 0.13, *r* = 0.28), or low-verbal false belief (*Z* = −1.31, *p* = 0.50, *r* = 0.24).

### Question 2: Are There Training Effects on the ToM Skills Other Than False Belief?

The children were tested also on True Complements, True Beliefs, and Precursors to False belief both pre and post training. There was no prediction that these would be affected by syntactic or lexical training, so a second set of analyses looked at the change in these variables compared to the variables targeted in Question 1.

The Box-Cox transformations were applied first to variables including precursors to false belief, true belief (verbal and low-verbal) and true complements, after which assumption of homogeneity of variance and normal distribution of residuals were met according to Levene’s tests and Shapiro’s tests.

In order to assess if the target training had a specific effect as compared to the control training on false complements and false belief attribution, both crucial to ToM, we ran factorial ANOVAs with the training group (syntax vs. lexical) and the moment of test (pre-test vs. post-test) as independent variables on the following dependent variables: true complements, verbal TB, low-verbal TB, and precursors to FB. The interaction effect between the training group (syntactic vs. lexical training) and the moment of test (pre-test vs. post-test) was not significant effect for true complements [*F*(1,55) = 0.58, *p* = 0.45], verbal TB [*F*(1,58) = 2.47, *p* = 0.12], low-verbal TB [*F*(1,58) = 2.03, *p* = 0.16], and precursors to FB [*F*(1,57) = 0.18, *p* = 0.67].

### Question 3: Are There Population Differences in the Effects of Training?

**FIGURE 6:**
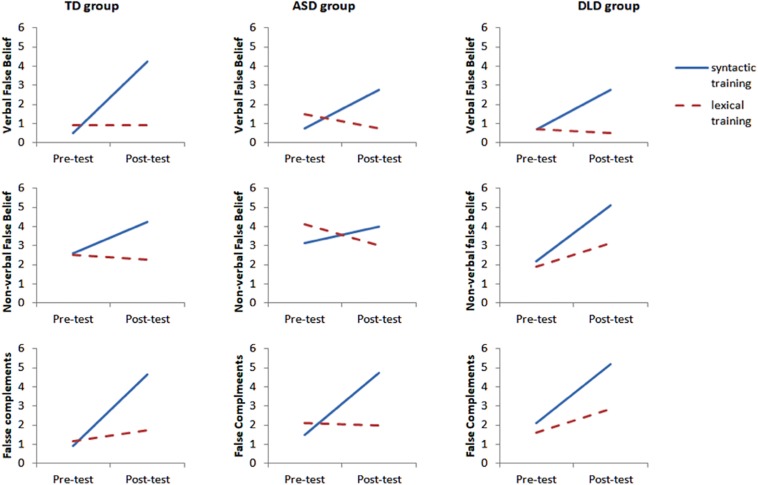
Interaction between training (syntax vs. lexicon) and moment of test (pre-test vs. post-test) on verbal false belief, low-verbal false belief and false complements for the three populations of children (TD, Typically Developing; ASD, Autism Spectrum Disorder; DLD, Developmental Language Disorder).

In order to compare the effects of the two trainings in the three populations, additional factorial ANOVAs were run (see Figure 6). This third ANOVA (time × training × clinical group) is exploratory, given the small sample sizes (approximately 10 children per condition). Results showed no interaction effect between training group (syntax vs. lexical), moment of test (pre-test vs. post-test) and population (TD vs. ASD vs. DLD) for false complement [*F*(2,105) = 0.54, *p* = 0.59], Verbal FB [*F*(2,103) = 0.56, *p* = 0.57], low-verbal FB [*F*(2,105) = 0.02, *p* = 0.98], true complements [*F*(2,105) = 1.12, *p* = 0.33], Verbal TB [*F*(2,108) = 0.60, *p* = 0.55], non-verbal TB [*F*(2,108) = 0.77, *p* = 0.46), and precursors to FB [*F*(2,106) = 0.68, *p* = 0.51].

A one-way ANOVA was conducted on the complement training condition only, with total post FB as the outcome and group as the independent variable. The untransformed data met the condition on homogeneity of variance by Levene’s test, and there was no significant difference between the three groups on total post-FB performance after complement training [*F*(2,27) = 0.546, *p* = 0.586].

### Question 4: Did the Lexical Training Group Show Differential Results on the Lexicon?

As for results on our standardized test of receptive lexicon, the syntactic training group did not show a difference between pre- and post-test scores (*Z* = 1.3, *p* = 0.2), whereas the lexical training group did (*Z* = 2.6, *p* < 0.01).^2^ For detailed information about participants and their individual results, see Supplementary Appendices C,D.

### Question 5: Did the Training Result Persist Beyond Immediate Post-test?

**FIGURE 7:**
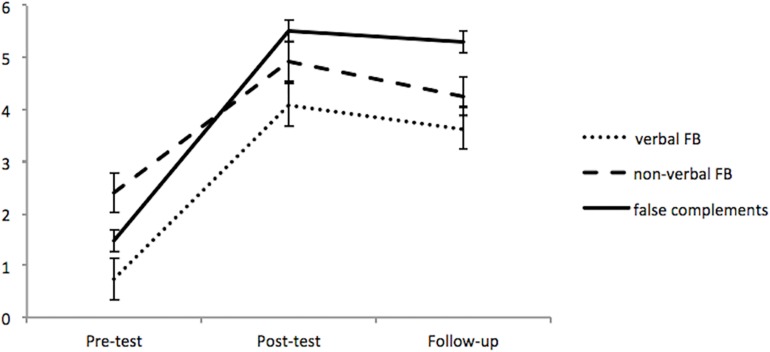
Scores on verbal FB, low-verbal FB and false complements at the moment of pre-test, post-test, and follow-up.

In order to assess if the progression observed at post-test was still present between pre-test and follow-up test, we ran non-parametric comparisons using Wilcoxon matched-pairs signed-ranks tests with the moment of test as the repeated variable (pre-test vs. follow-up and post-test versus follow-up) for the dependent variables, on 22 children who had showed gains of at least 10% on the post-test in the syntactic training group. The progression between pre-test and follow-up test was statistically significant for verbal FB (*p* = 0.001, *r* = 0.81), low-verbal FB (*p* = 0.003, *r* = 0.64), and false complements (*p* = 0.001, *r* = 0.88), with higher scores in follow-up. The mean scores were higher on immediate post-tests compared to follow-up post-tests, but the difference between these two post-tests was not significant for verbal FB (*p* = 0.265, *r* = 0.035), and did not reach significance for low-verbal FB (*p* = 0.066, *r* = 0.39) or false complements (*p* = 0.096, *r* = 0.36). Thus there was generally only a small drop between post-test and follow-up 4–6 weeks after the first post-test (see Figure 7).

### Question 6: What Is the Precise Effect of Training on False Complements?

More powerful statistics were used to explore the contributing effects of the background variables and training conditions. Although certain criteria had to be met to be included in the study, it would be impossible with such a small sample to match the groups on every variable. Using multiple regressions first to discover which variables share variance with the outcome, structural equation modeling (SEM) provided a powerful tool with which to look at the paths of influence on the outcome of false belief understanding.^3^ For example, did the initial level of False Belief understanding contribute to the training effect? What about non-verbal intelligence, as measured by Ravens, or the level of general language skill (using EXALANG)? And did the child’s trained mastery of false complements contribute to the false belief post-test score, or did some children succeed on post-test even if they did not improve on complements?

The ANOVAs across training and control groups showed no difference across the TD, ASD and DLD populations in the outcome, nor any interactions between populations and training groups. For that reason, the groups and populations could be collapsed to explore regressions with the outcome variable of false beliefs (combining low-verbal and verbal tasks into one 12-point score). The final regressions contained just the variables that contributed unique variance to this outcome. As shown in Table 3, first age was entered and then Raven’s matrices, the total of pre-training score on False belief (non-verbal and verbal combined), then Training condition, then the total post-training score on False complements, since both of these variables contributed to the outcome.

**TABLE 3:** Regression results predicting children’s total post-false belief (*N* = 60).

**Predictor**	***B***	***B* 95% CI [LL, UL]**	***SE B***	**β**	***sr*^2^**	***r***	**Fit**	**Difference**
(Intercept)	–0.78	[−3.74, 2.18]	1.48					
Age	–0.36	[−0.75, 0.04]	0.20	–0.19	0.03	–0.04		
Raven’s Total	0.26^∗∗^	[0.09, 0.42]	0.08	0.31	0.08	0.26^∗^		
Total Pre-False Belief	0.61^∗∗^	[0.28, 0.93]	0.16	0.35	0.12	0.30^∗^		
Training	4.88^∗∗∗^	[3.38, 6.39]	0.75	0.60	0.35	0.58^∗∗^		
							*R^2^* = 0.547^∗∗^	
(Intercept)	–1.08	[−3.65, 1.48]	1.28					
Age	–0.32	[−0.67, 0.02]	0.17	–0.17	0.02	–0.04		
Raven’s Total	0.19^∗^	[0.04, 0.34]	0.07	0.23	0.04	0.26^∗^		
Total Pre-False Belief	0.51^∗∗∗^	[0.22, 0.79]	0.14	0.30	0.08	0.30^∗^		
Training	2.50^∗∗^	[0.81, 4.19]	0.84	0.31	0.05	0.58^∗∗^		
Total Post-False Complements	0.79^∗∗∗^	[0.43, 1.15]	0.18	0.46	0.12	0.72^∗∗^		
							*R*^2^ = 0.667^∗∗^	Δ*R*^2^ = 0.120^∗∗^

*A significant *B*-weight indicates the beta-weight and semi-partial correlation are also significant. *B* represents unstandardized regression weights. β indicates the standardized regression weights. *sr*^2^ represents the semi-partial correlation squared. *r* represents the zero-order correlation. *LL* and *UL* indicate the lower and upper limits of a confidence interval, respectively. ^∗^*p* < 0.05. ^∗∗^*p* < 0.01. ^∗∗∗^*p* < 0.001.*

**FIGURE 8:**
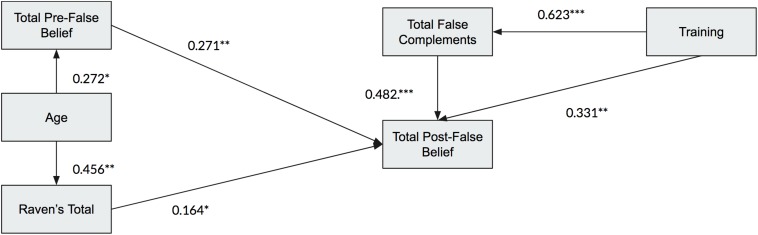
Standardized parameter estimation for the hypothesized model.

Having established the significant variables in the regression for prediction of the outcome, various SEM models were tried to find the model with the best fit. Although the number of subjects is on the low side for a SEM, the fit indices can give an indication of whether the sample has sufficient power to justify the model. Table 4 shows the results, and Figure 8 shows the optimum model result, with excellent fit indices.

**TABLE 4:** Standardized parameter estimates for the hypothesized model (*N* = 60).

	***B***	***SE***	**β**	***p***
**Path Analysis**				
Raven’s Total → Total Post-FB	0.13^∗^	0.06	0.16	0.040
Total Pre-False Belief → Total Post-FB	0.45^∗∗∗^	0.13	0.27	0.001
Training → Total Post-FB (c)	2.63^∗∗∗^	0.80	0.33	0.001
Total Post-False Complements → Total Post-FB (b)	0.80^∗∗∗^	0.17	0.48	0.000
Age → Raven’s Total	1.05^∗∗^	0.27	0.46	0.000
Age → Total Pre-False Belief	0.28^∗^	0.14	0.26	0.038
Training → Total Post-False Complements (a)	3.00^∗∗∗^	0.48	0.62	0.000
**Indirect Effect**				
a × b	2.38^∗∗∗^	0.63	0.30	0.001
**Total Effect**				
c	5.01^∗∗∗^	0.74	0.63	0.000

*Standardized parameter estimation for the hypothesized model. All reported estimates are the maximum likelihood standardized point estimates. □2(7, *N* = 60) = 8.934, *p* = 0.257; comparative fit index = 0.982; Tucker-Lewis index = 0.963, root mean square error of approximation = 0.068, standardized root mean square residual = 0.067. ^∗^*p* < 0.05. ^∗∗^*p* < 0.01. ^∗∗∗^*p* < 0.001.*

As background variables, the Raven’s score and the pre-test False belief score contributed to the final outcome, but age did not have a direct effect. Importantly, the training condition had a significant effect on both the False Belief outcome, and also on the children’s skill on the False complement post-task. That skill then contributed significantly also to False belief. A further analysis asked whether the Training on False complements was instrumental in the outcome on False Belief via two paths: one direct, and the other with final performance on False Complements as the mediating variable. The analysis revealed that there was indeed an additional mediating effect of the false complements, as revealed in Table 4. Training has a highly significant effect on complements (a), which then has an effect on False Beliefs (b). So the *indirect* effect is a x b. In addition, Training has a *direct* effect (c) on false beliefs, also highly significant.

## Discussion

Theory of Mind plays a fundamental role in social cognition (Harris, 2006), and an important step of ToM development occurs around the age of 4–5 years, when TD children begin to understand that others have beliefs that differ from their own and may be in conflict with reality (Wellman et al., 2001). In contrast, marked delays in consolidating this step in mental reasoning can be observed in children with ASD (Yirmiya et al., 1998), and more subtle delays in children with DLD (Nilsson and de López, 2016). The overarching objective of this work was to identify an efficient way to address ToM difficulties in ASD and DLD.

Amidst these clinical populations, the subset succeeding at tasks assessing false beliefs has been shown to display a better level of language, and in particular grammatical skills (ASD: Fisher et al., 2005; Paynter and Peterson, 2010; DLD: Farrar et al., 2009; Andrés-Roqueta et al., 2013). Some authors claim that mastery of ‘complement clauses’ such as ‘X thinks/ says that Y,’ would be the grammatical component *par excellence* facilitating belief reasoning (de Villiers, 2000, 2007), including in ASD and DLD (Tager-Flusberg and Joseph, 2005; Durrleman et al., 2016a, 2017a), because the content of the embedded sentence may refer to a subjective truth. In light of the fact the privileged links have been found between complements and belief reasoning, not only in TD (de Villiers and Pyers, 2002) but also in clinical populations including ASD and DLD (Farrar et al., 2017), our main objective with this work was to see if complementation training could yield similar gains across these populations. As such, our target program aimed to enhance complementation so as to boost ToM performance not only in TD, which has already been found, but also in ASD and DLD, which has never before been investigated. This training was compared to a control training, which promoted lexical enrichment. Our findings replicate the results for TD children in other languages to French indicating that syntactic training focussing on sentential complements improves both these structures as well as performance on false belief attribution in this population (Hale and Tager-Flusberg, 2003; Lohmann and Tomasello, 2003; Shuliang et al., 2014). Moreover, children with DLD and ASD, who can display delays in both syntax and ToM (Yirmiya et al., 1998; Nilsson and de López, 2016), did not show any difference from the TD group regarding these positive outcomes. This first attempt at explicit syntactic training with a group including these clinical populations is thus encouraging regarding the potential direct linguistic gains as well as the indirect cognitive benefits, as measured by ToM tasks.

The ToM benefits associated with enhanced complementation skills were observed specifically for false belief reasoning, and not, e.g., for precursors of this ability such as the comprehension of diverse beliefs and desires (Wellman and Liu, 2004) or true belief items which can be resolved via reality responses. The interest of complementation appears indeed to support a specific component of ToM. It is important to underline that improvement on FB was observed whether measured via verbal or low-verbal tasks. Performance on both of these measures was indeed correlated to complementation skills, suggesting that language supports not only FB-task performance, but also the *reasoning* implied in belief attribution (de Villiers, 2007). It is also interesting to note that both general grammatical skills and specific mastery of complementation relate to mentalizing abilities in typical development on post-tests, while atypical development appears to specifically capitalize on complementation, in line with previous reports (Farrar et al., 2017). This may suggest different pathways to FB understanding, potentially related to differences between these populations to benefit from social interactions (Farrar et al., 2017).

Encouragingly, improvements in complements and ToM were observed not only during immediate tests but also in follow-up post-tests, revealing that the training effects were still detectible over time as revealed by higher performance on follow-up post-tests compared to pre-tests. Still, despite this durability in improvements between pre-tests and post-tests conducted roughly a month after training ceased, there was nevertheless a very modest drop in performance between immediate post-tests and follow-up post-tests, which could indicate that more training would be required for the results to be sustainable. None of the previous studies examining the effects of complementation training in TD children included follow-up post-tests (Hale and Tager-Flusberg, 2003; Lohmann and Tomasello, 2003; Shuliang et al., 2014), thus more work is needed to determine whether or not this is specific to our cohort which included clinical children.

While the children of all populations (TD, DLD, and ASD) who received the control training improved their lexical abilities, they did not improve skills on complements or ToM. The increase in performance observed in the target-training group on both complements and FB is therefore not achievable via just any linguistic stimulation, but rather stems from a specific training on complementation, here administered via DIRE.

The training on complements had two effects, one direct and one indirect via the improvement on children’s own complementation skills. That is, some component of the outcome variance was contributed by being exposed to an enhanced and carefully designed verbal input on complementation, and the other, by the child’s skill in producing correct answers to complementation questions. Evidence for both effects is found in the developmental literature on false beliefs. Research has shown that families that engage in much discourse about mental states, which frequently is coupled with complements given the argument structure of cognitive verbs, have children who develop false belief skills earlier (Tompkins et al., 2018). Much debate has arisen over whether the causal effect is on the child’s own language, or directly in providing evidence for the theory the child is building about other minds (de Villiers and de Villiers, 2014). The model in this experimental study hints at a role for each effect.

Future work on the effects of complementation training on ToM should seek to include larger cohorts of children with DLD and ASD, as well as other populations such as deaf children, who also show difficulties with both embedding (Tuller and Delage, 2014) and ToM (Peterson and Siegal, 2000). Subsequent studies should also seek to determine whether or not the gains are limited in scope (e.g., giving rise merely to verbal strategies for solving ToM tasks, see e.g., Leslie and Roth, 1993; Happé, 1995; Tager-Flusberg etal., 1997; Tager-Flusberg, 2000; Senju et al., 2010) or short-term, as these are important concerns, especially for children on the autism spectrum. It would thus be worthwhile to include a dimension of testing involving more ecological tasks, such as hide and seek, as well as parent questionnaires assessing the quality of the children’s social interactions, testing to be also conducted well after the intervention has ceased. This would allow a deeper understanding of whether children’s enhanced grasp of belief reasoning resulting from complementation training can give rise to more successful social skills, including in the long term. Such results could provide clearer arguments in favor of the benefits of syntactic remediation in ToM programs across aetiologies.

## Data Availability Statement

The datasets generated for this study are available on request to the corresponding author.

## Author Contributions

SD designed the study in collaboration with HD as well as JD and ET. SD, HD, and ET orchestrated recruiting and training. MB and RY analyzed data, with RY more specifically building structural equation models and fine-tuning results. SD wrote the manuscript with the input from HD, JD, ET, and MB.

## Conflict of Interest

The authors declare that the research was conducted in the absence of any commercial or financial relationships that could be construed as a potential conflict of interest.

## Footnotes

1 A total of 11 children were eliminated after the pre-tests from the initial cohort of 71 participants: 3 TD, 4 ASD and 4 DLD. For 10 children, this was because of ceiling performance, and for 1 child with ASD this was because of hypersensitivity to the sounds of the testing material.

2 One child with ASD in the lexical training group displayed particularly poor performance in lexical designation in the pre-test, which could bias group results in the comparison between pre- and post-test. However, the progression of the “lexical training group” is still significant even when removing this child from the analyses (*Z* = 2.4, *p* < 0.05). Note also that we proceeded to non-parametrical (Wilcoxon signed-rank) tests for these comparisons, due to ceiling effects on this measure.

3 A 10:1 ratio of cases to free parameters is suggested as a minimum sample size for SEM by Bentler and Chou (1987) and echoed by many others as a rule of thumb. We test six parameters with a sample of 60 participants. Though the 10:1 ratio is often considered safe, there are simulations (e.g., Nevitt and Hancock, 2004; Wolf et al., 2013) that suggest higher numbers are desirable in certain conditions. Latent variables would render the sample size insufficient compared to the simple path model explored here. Missing data also would drastically increase the necessary sample size. An insufficient sample size can result in a failure to converge on a model, or have poor fit estimates. The current model does not show these problems, and is supported by the regressions, so we judge it to be a worthwhile addition despite the small sample.

## References

[B1] Alzrayer N. Banda D. R. Koul R. K. (2014). Use of iPad/iPods with individuals with autism and other developmental disabilities: a meta-analysis of communication interventions. *Rev. J. Autism Dev. Disord.* 1 179–191. 10.1007/s40489-014-0018-5

[B2] American Psychiatric Association [APA] (2000). *Diagnostic and Statistical Manual of Mental Disorders*, 4th Edn Washington, DC: American Psychiatric Association.

[B3] Andrés-Roqueta C. Adriàn J. E. Clemente R. A. Katsos N. (2013). Which are the best predictors of Theory of Mind delay in children with specific language impairment? *Int. J. Lang. Commun. Disord.* 48 726–737. 10.1111/1460-6984.12045 24165368

[B4] Astington J. W. (2003). “Sometimes necessary, never sufficient: false belief understanding and social competence,” in *Individual Differences in Theory of Mind : Implications for Typical and Atypical Development*, eds Repachol B. Slaughter V., (New York, NY: Psychology Press), 13–38.

[B5] Astington J. W. Edward M. J. (2010). Language matters for theory of mind. *Int. Soc. Study Behav. Dev. Newsletter* 45 7–9.

[B6] Astington J. W. Jenkins J. M. (1999). A longitudinal study of the relation between language and theory-of-mind development. *Dev. Psychol.* 35 1311–1320. 10.1037/0012-1649.35.5.1311 10493656

[B7] Astington J. W. Pelletier J. (2005). “Theory of mind, language, and learning in the early years: developmental origins of school readiness,” in *The development of social cognition and communication*, eds Homer B. D. Tamis-Lemonda C. S., (Mahwah, NJ: Erlbaum), 205–230.

[B8] Baron-Cohen S. (1990). Autism: a specific cognitive disorder of ’mind-blindness. *Int. Rev. Psychiatry.* 2 81–90. 10.3109/09540269009028274

[B9] Baron-Cohen S. Leslie A. Frith U. (1985). Does the autistic child have a “theory of mind”? *Cognition* 21 37–46. 10.1016/0010-0277(85)90022-8 2934210

[B10] Bentler P. M. Chou C. H. (1987). Practical issues in structural modeling. *Sociol. Methods Res.* 16 78–117. 10.1177/0049124187016001004

[B11] Bishop D. (2013). Problems with tense marking in children with specific language impairment: not how but when. *Phil. Trans. R. Soc. B Biol. Sci.* 369:20120401. 10.1098/rstb.2012.0401 24324242 PMC3866428

[B12] Bloom L. Rispoli M. Gartner B. Hafitz J. (1989). Acquisition of complementation. *J. Child Lang.* 16 101–120. 10.1017/s0305000900013465 2925807

[B13] Burnel M. Perrone-Bertolotti M. Reboul A. Baciu M. Durrleman S. (2017). Reducing the language content in tom tests: a developmental scale. *Dev. Psychol*. 54 293–307. 10.1037/dev0000429 29154651

[B14] Cheung H. Hsuan-Chih C. Creed N. Ng L. Wang S. P. Mo L. (2004). Relative roles of general and complementation language in theory-of-mind development: evidence from cantonese and english. *Child Dev.* 75 1155–1170. 10.1111/j.1467-8624.2004.00731.x 15260870

[B15] Chevallier C. Parish-Morris J. Tonge N. Le L. Miller J. Schultz R. T. (2014). Susceptibility to the audience effect explains performance gap between children with and without autism in a theory of mind task. *J. Exp. Psychol.* 143: 972. 10.1037/a0035483 24392710 PMC4038654

[B16] Cohen J. (1988). *Statistical Power Analysis for the Behavioral Sciences*, 2nd Edn Hillsdale, NJ: Erlbaum.

[B88] De La Santé O. M. (1993). *CIM 10–Classification Internationale des Troubles Mentaux et des Troubles du Comportement: Descriptions Cliniques et Directives Pour le Diagnostic.* Paris: Masson.

[B17] de Villiers J. (2000). “Language and theory of mind. what are the developmental relationships,” in *Understanding Other Minds: Perspectives from Developmental Cognitive Neuroscience*, eds Baron-Cohen S. Tager-Flusberg H. Cohen D. J., (New York, NY: Oxford University Press), 83–123.

[B18] de Villiers J. (2007). The interface of language and theory of mind. *Lingua* 117 1858–1878. 10.1016/j.lingua.2006.11.006 17973025 PMC2000852

[B19] de Villiers J. de Villiers P. (2000). “Linguistic determination and the understanding of false beliefs,” in *Children’s Reasoning and the Mind*, eds Mitchell P. Riggs K. J., (Hove: Psychology Press), 191–228.

[B20] de Villiers J. de Villiers P. (2014). The role of language in theory of mind development. *Top Lang. Disord.* 34 313–328. 10.1097/tld.0000000000000037

[B21] de Villiers J. Hobbs K. Hollebrandse B. (2014). “Recursive complements and propositional attitudes,” in *Recursion: Complexity in Cognition*. Heidelberg, eds Speas M. Roeper T., (New York, NY: Springer), 221–242. 10.1007/978-3-319-05086-7_10

[B22] de Villiers J. G. Pyers J. (2002). Complements to cognition: a longitudinal study of the relationship between complex syntax and false belief understanding. *Cogn. Dev.* 17 1037–1060. 10.1016/s0885-2014(02)00073-4

[B23] de Villiers P. A. Burns F. Pearson B. Z. (2003). “The role of language in the theory of mind development of language-impaired children: complementing theories,” in *Proceedings of the 27th Annual Boston University Conference on Language Development*, eds Beachley B. Brown A. Conlin F., (Somerville, MA: Cascadilla Press), 232–242.

[B24] Dennett D. (1978). Beliefs about beliefs. *Behav. Brain Sci.* 4 568–570.

[B25] Derksen D. G. Hunsche M. C. Giroux M. E. Connolly D. A. Bernstein D. M. (2018). A systematic review of theory of mind’s precursors and functions. *Zeitschrift Psychol.* 226 87–97. 10.1027/2151-2604/a000325

[B26] Diessel H. (2004). *The Acquisition of Complex Sentences.* Cambridge: Cambridge University Press.

[B27] Durrleman S. Burnel M. Reboul A. (2017a). Theory of mind in SLI revisited: links with syntax, comparisons with ASD. *Int. J. Lang. Commun. Disord.* 52 816–830. 10.1111/1460-6984.12317 28470886

[B28] Durrleman S. Burnel M. Thommen E. Foudon N. Sonié S. Reboul A. (2016a). The language-cognition interface in ASD: complement sentences and false belief reasoning. *Res. Autism Spectrum Disord.* 21 109–120. 10.1016/j.rasd.2015.10.003

[B29] Durrleman S. Da Costa J. Delage H. (2016b). *Différencier l’Idée de la Réalité par Exercices (DIRE).* Geneva: University of Geneva.

[B30] Durrleman S. Franck J. (2015). Exploring links between language and cognition in autism spectrum disorders: complement sentences, false belief, and executive functioning. *J. Commun. Disord.* 54 15–31. 10.1016/j.jcomdis.2014.12.001 25637130

[B31] Durrleman S. Gatignol P. Delage H. (2017b). *Can Theory of Mind be Improved Thanks to Grammatical Training? A Study of Children with Autism Spectrum Disorder and Specific Language Impairment (‘La Théorie de l’esprit peut-elle s’améliorer grâce à un Entraînement Grammatical? Une étude chez les enfants Atteints de Troubles du Spectre Autistique et de Troubles Spécifiques du Langage’).* Paris: Actes du XVII° Congrès de L’Union Nationale pour le Développement de la Recherche et de l’Evaluation en Orthophonie (UNADREO).

[B32] Farrar M. J. Benigno J. P. Tompkins V. Gage N. A. (2017). Are there different pathways to explicit false belief understanding? General language and complementation in typical and atypical children. *Cogn. Dev.* 43 49–66. 10.1016/j.cogdev.2017.02.005

[B33] Farrar M. J. Johnson B. Tompkins V. Easters M. Zilisi-Medus A. Benigno J. P. (2009). Language and theory of mind in preschool children with specific language impairment. *J. Commun. Disord.* 42 428–441. 10.1016/j.jcomdis.2009.07.001 19647837

[B34] Fisher N. Happé F. Dunn J. (2005). The relationship between vocabulary, grammar, and false belief task performance in children with autistic spectrum disorders and children with moderate learning difficulties. *J. Child Psychol. Psychiatr.* 46 409–419. 10.1111/j.1469-7610.2004.00371.x 15819650

[B35] Flavell J. H. (1999). Cognitive development: children’s knowledge about the mind. *Annu. Rev. Psychol.* 50 21–45. 10.1146/annurev.psych.50.1.21 10074674

[B36] Forgeot d’Arc B. Ramus F. (2011). Belief attribution despite verbal interference. *Q. J. Exp. Psychol.* 64 975–990. 10.1080/17470218.2010.524413 21161856

[B37] Gefen D. Straub D. Boudreau M. (2000). Structural equation modeling and regression: guidelines for research practice. *Commun. Assoc. Inform. Syst.* 4 2–76. 10.17705/1CAIS.00407

[B38] Gopnik A. Astington J. W. (1988). Children’s understanding of representational change and its relation to the understanding of false belief and the appearance-reality distinction. *Child Dev.* 59 26–37. 10.1111/j.1467-8624.1988.tb03192.x 3342716

[B39] Hale C. M. Tager-Flusberg H. (2003). The influence of language on theory of mind: a training study. *Dev. Sci.* 6 346–359. 10.1111/1467-7687.00289 16467908 PMC1350918

[B40] Happé F. G. E. (1995). The role of age and verbal ability in the theory of mind task performance of subjects with autism. *Child Dev.* 66 843–855. 10.1111/j.1467-8624.1995.tb00909.x 7789204

[B41] Harris P. L. (2006). “Social cognition,” in *Cognition, perception, and language*, 6th Edn, eds Kuhn D. Siegler R. S., (Hoboken, NJ: Wiley).

[B42] Helloin M.-C. Thibault M.-P. (2006). *l’EXALANG 3-6.* Mont-Saint: Ortho-Mothus.

[B43] Holmes A. M. (2002). Theory of mind and behavior disorders in children with specific language impairment [abstract]. *Diss. Abstr. Intern.: Sec. B Sci. Eng.* 62 1–136.

[B44] Leonard L. B. (2014). *Children with Specific Language Impairment.* Cambridge, MA: MIT press.

[B45] Leslie ‘A. M. Roth D. (1993). “What autism teaches us about metarepresentation,” in *Understanding Other Minds: Perspectives From Autism*, eds BaronCohen S. TagerFlusberg H. Cohen D. J. (Oxford: Oxford University Press), 83–111.

[B46] Lind S. E. Bowler D. M. (2009). Language and theory of mind in autism spectrum disorder: the relationship between complement syntax and false belief task performance. *J. Autism Dev. Disord.* 39 929–937. 10.1007/s10803-009-0702-y 19205856

[B47] Lohmann H. Tomasello M. (2003). The role of language in the development of false belief understanding: a training study. *Child Dev.* 74 1130–1144. 10.1111/1467-8624.00597 12938709

[B48] Lord C. Rutter M. DiLavore P. C. Risi S. (2003). *Autism Diagnostic Observation Schedule: Manual.* Los Angeles, CA: Western Psychological Services.

[B49] Mazza M. Mariano M. Peretti S. Masedu F. Pino M. C. Valenti M. (2017). The role of theory of mind on social information processing in children with autism spectrum disorders: a mediation analysis. *J. Autism Dev. Disord.* 47 1369–1379. 10.1007/s10803-017-3069-5 28213839

[B50] Miller C. A. (2001). False belief understanding in children with specific language impairment. *J. Commun. Disord.* 34 73–86. 10.1016/s0021-9924(00)00042-3 11322571

[B51] Miller C. A. (2004). False belief and sentence complement performance in children with specific language impairment. *Intern. J. Lang. Commun. Disord.* 39 191–213. 10.1080/13682820310001616994 15204451

[B52] Milligan K. Astington J. W. Dack L. A. (2007). Language and theory of mind: meta-analysis of the relation between language ability and false-belief understanding. *Child Dev.* 78 622–646. 10.1111/j.1467-8624.2007.01018.x 17381794

[B53] Nevitt J. Hancock G. R. (2004). Evaluating small sample approaches for model test statistics in structural equation modeling. *Multivar. Behav. Res.* 39 439–478. 10.1207/s15327906mbr3903_3

[B54] Nilsson K. K. de López J. (2016). Theory of mind in children with specific language impairment : a systematic review and meta-analysis. *Child Dev.* 87 143–153. 10.1111/cdev.12462 26582261

[B55] Paynter J. Peterson C. C. (2010). Language and ToM development in autism versus asperger syndrome: contrasting influences of syntactic versus lexical/semantic maturity. *Res. Autism Spectrum Disord.* 4 377–385. 10.1016/j.rasd.2009.10.005

[B56] Paynter J. Peterson C. C. (2013). Further evidence of benefits of thought-bubble training for theory of mind development in children with autism spectrum disorders. *Res. Autism Spectrum Disord.* 7 344–348. 10.1016/j.rasd.2012.10.001

[B57] Perner J. (1988). “Developing semantics for theories of mind: from propositional attitudes to mental representation,” in *Developing Theories of Mind*, eds Astington J. W. Harris P. L. Olson D. R., (Cambridge: Cambridge University Press), 141–172.

[B58] Perner J. Sprung M. Zauner P. Haider H. (2003). Want that is understood well before say that, think that, and false belief: a test of de villiers’ linguistic determinism on German-speaking children. *Child Dev.* 74 179–188. 10.1111/1467-8624.t01-1-00529 12625444

[B59] Peterson C. C. Siegal M. (2000). Insights into a theory of mind from deafness and autism. *Mind Lang.* 16 77–99.

[B60] Premack D. Woodruff G. (1978). Does the chimpanzee have a theory of mind? *Behav. Brain Sci.* 1 515–526. 10.1017/s0140525x00076512

[B61] Prior M. R. Dahlstrom B. Squires T. L. (1990). Autistic children’s knowledgeof thinking and feeling states in other people. *J. Child Psychol. Psychiatry* 31 587–601. 10.1111/j.1469-7610.1990.tb00799.x 2365761

[B62] Raven J. Raven J. C. Court J. H. (1998). *Raven Manual: Section 4, Advanced Progressive Matrices.* Oxford: Oxford Psychologists Press Ltd.

[B63] Rice M. L. Wexler K. (1996). Toward tense as a clinical marker of specific language impairment in English-speaking children. *J. Speech Hear. Res.* 39 239–257. 8959609 10.1044/jshr.3906.1239

[B64] Roberts J. A. Rice M. L. Tager-Flusberg H. (2004). Tense marking in children with autism. *Appl. Psychol.* 25 429–448. 10.1017/s0142716404001201

[B65] Rosenthal R. (1994). “Parametric measures of effect size,” in *The Handbook of Research Synthesis*, eds Cooper H. Hedges L. V., (New York, NY: Russell Sage Foundation), 231–244.

[B66] Rutter M. Le Couteur A. Lord C. (2003). *ADI-R. Autism Diagnostic Interview Revised.* Manual, LA: Western Psychological Services.

[B67] Schick B. De Villiers P. De Villiers J. Hoffmeister R. (2007). Language and theory of mind: a study of deaf children. *Child Dev.* 78 376–396. 10.1111/j.1467-8624.2007.01004.x 17381779

[B68] Senju A. Southgate V. Miura Y. Matsui T. Hasegawa T. Tojo Y. (2010). Absence of spontaneous action anticipation by false belief attribution in children with autism spectrum disorder. *Dev. Psychopathol.* 22 353–360. 10.1017/S0954579410000106 20423546

[B69] Shuliang M. Yanjie S. Sabbagh M. A. (2014). Sentential complements and false belief understanding in chinese mandarin-speaking preschoolers: a training study. *Cogn. Dev.* 29 50–61. 10.1016/j.cogdev.2013.11.001

[B70] Steel G. Rose M. Eadie P. (2016). The production of complement clauses in children with language impairment. *J. Speech Lang. Hear. Res.* 59 330–341. 10.1044/2015_jslhr-l-15-0001 27089061

[B71] Tager-Flusberg H. (1989). “A psycholinguistic perspective on language development in the autistic child,” in *Autism: New Directions in Diagnosis, Nature and Treatment*, ed. Dawson G., (New York, NY: Guilford Press), 92–115.

[B72] Tager-Flusberg H. (2000). “Language and understanding minds: Connections in autism,” in *Understanding Other Minds: Perspectives From Autism and Developmental Cognitive Neurosciences*, eds Baron-Cohen S. Tager-Flusberg H. Cohen D., (Oxford: Oxford University).

[B73] Tager-Flusberg H. Joseph R. M. (2005). “How language facilitates the acquisition of false-belief understanding in children with autism,” in *Why Language Matters for Theory of Mind*, eds Astington J. W. Baird J. A., (New York, NY: Oxford University Press), 298–318. 10.1093/acprof:oso/9780195159912.003.0014

[B74] Tager-Flusberg H. Sullivan K. Boshart J. (1997). Executive functions and performance on false belief tasks. *Dev. Neuropsychol.* 13 487–493. 10.1080/87565649709540689

[B75] Tardif T. Wing-Chee S. C. Kaciroti N. (2007). Language and false belief: evidence for general, not specific, effects in cantonese-speaking preschoolers. *Dev. Psychol.* 43 318–340. 10.1037/0012-1649.43.2.318 17352542

[B76] Tompkins V. Benigno J. Kiger Lee B. Wright B. (2018). The relation between parents’ mental state talk and children’s social understanding: a meta-analysis. *Soc. Dev.* 27 223–246. 10.1111/sode.12280

[B77] Tucker L. (2004). *Specific Language Impairment and Theory-of-Mind: Is Normal Language Development an Essential Precursor for on Time Theory-of-Mind Development.* Crawley WA: University of Western Australia.

[B78] Tuller L. Delage H. (2014). Mild-to-moderate hearing loss and language impairment: how are they linked? *Lingua* 139 80–101. 10.1016/j.lingua.2013.10.009

[B79] Tuller L. Henry C. Sizaret E. Barthez M. A. (2012). Specific language impairment at adolescence: avoiding complexity. *Appl. Psychol.* 33 161–184. 10.1017/s0142716411000312

[B80] Wellman H. Cross D. Watson J. (2001). Meta-analysis of theory of mind development: the truth about false belief. *Child Dev.* 72 655–684. 10.1111/1467-8624.00304 11405571

[B81] Wellman H. M. Baron-Cohen S. Caswell R. Gomez J. C. Swettenham J. Toye E. (2002). Thought-bubbles help children with autism acquire an alternative to a theory of mind. *Autism* 6 343–363. 10.1177/1362361302006004003 12540127

[B82] Wellman H. M. Liu D. (2004). Scaling of theory-of-mind task. *Child Dev.* 75 523–541. 10.1111/j.1467-8624.2004.00691.x 15056204

[B83] Wilson M. S. Fox B. J. (2013). *Language for Theory of Mind: Understanding Others’ Perceptions, Wants, and Needs.* Winooski, VT: Laureate Learning Systems.

[B84] Wimmer H. Perner J. (1983). Beliefs about beliefs: representation and constraining function of wrong beliefs in young children’s understanding of deception. *Cognition* 13 103–128. 10.1016/0010-0277(83)90004-5 6681741

[B85] Wolf E. J. Harrington K. M. Clark S. L. Miller M. W. (2013). Sample size requirements for structural equation models: an evaluation of power, bias, and solution propriety. *Educ. Psychol. Measure.* 73 913–934. 25705052 10.1177/0013164413495237 PMC4334479

[B86] Woolfe T. Want S. C. Siegal M. (2002). Signposts to development: theory of mind in deaf children. *Child Dev.* 73 768–778. 10.1111/1467-8624.00437 12038550

[B87] Yirmiya N. Erel O. Shaked M. Solomonica-Levi D. (1998). Meta-analyses comparing theory of mind abilities of individuals with autism, individuals with mental retardation, and normally developing individuals. *Psychol. Bull.* 124 283–307. 10.1037/0033-2909.124.3.283 9849110

