# Supplementary material for: The Impact of Grammar on Mentalizing: A Training Study Including Children With Autism Spectrum Disorder and Developmental Language Disorder
Source: Front Psychol. 2019 Nov 19;10:2478. doi: 10.3389/fpsyg.2019.02478 (PMC6877902; doi:10.3389/fpsyg.2019.02478)
Supplement: Supplementary file 1 [file Data_Sheet_1.pdf]

Appendix A

The first activity was inspired by the Laureate Learning programme[1] (Wilson & Fox, 2013) and focused on complements with an infinitival verb such as *Sophie voit un bébé en train de rire et Anna voit un bébé en train de pleurer* ("Sophie sees a baby laughing and Anna sees a baby crying"). We wanted to ensure that these were consolidated as they precede tensed complements sentences in language development (Bloom, Rispoli, Gartner, and Hafitz, 1989; Diessel, 2004). The child's comprehension of these sentences was assessed via questions such as: "Who sees a baby laughing?" and in the event of a non-response or incorrect answer, the question was repeated along with an indication of the correct answer by means of highlighting the accurate image (i.e. of Sophie watching a baby laughing). The entire procedure was repeated later on in the training protocol allowing the child the chance of an independent response.

All other activities focused on tensed complements of communication verbs. In the second activity, the child heard an embedded sentence introduced by a tensed verb, such as: "The little girl screams that there is a spider in the bathtub". In half of the cases, what is reported corresponds to reality (i.e. there was indeed a spider) while in the other half, it was in conflict with reality (e.g. there was a stain which may resemble a spider). The child was asked: "What did the little girl scream?" (Figure i). At this point, the child had to select the content of the complement. In the case of a mistake, the relevant response was again indicated and the child was offered an explanation, e.g. "It's only a stain, but the little girl screamed there was a spider. What did the little girl scream? She screamed that it was a spider".

[1] We more specifically capitalized on modules 4-6 of the 'Language for Theory of Mind' CD-Rom.

Supplementary Figure S1: Examples of activity 2, DIRE

The third activity was based on the training protocol of Hale & Tager-Flusberg (2003), where characters comment on activities they are doing, but their commentary is not always accurate. In our examples, this was made plausible because the characters could not clearly assess the situation, due to having their eyes closed or looking another way. For example, children see a girl, Sarah, reaching beneath her chair and a voice comments: "Look, I'm petting the dog". In one scenario, this commentary corresponds to what is really happening (because the dog is really beneath the chair) while in others it does not (because in fact the cat has slipped under the chair). The child then heard a question: “What did Sarah say?” and had to select between two images, one with Sarah accompanied by a speech bubble of herself petting a dog, another showing Sarah accompanied by a speech bubble of herself petting a cat. Children had to point to the image which reflected the contents of the complement (so containing the dog). In instances when they were right, they heard: “Yes, that’s right! Sarah said that she was petting the dog, but in fact Sarah was petting the cat”. In instances of errors, for example here a speech bubble containing the cat, children received corrective feedback: “Remember, Sarah said she was petting the dog, but actually she was petting the cat”. Finally, children were systematically asked to repeat the target, complement structure, in both correct and incorrect designations: “Can you repeat? Sarah said she was petting the dog, but actually she was petting the cat”.

The fourth activity involved different levels of complexity. At the first level, children were encouraged to distinguish simple matrix sentences such as "There is a hedgehog on the ground", from those corresponding to embedded complements, such as "The boy says there is a hedgehog on the ground". This involved selecting either a picture of a hedgehog or of a boy accompanied by a speech bubble containing a hedgehog. At the second level, children had to distinguish between two complements, such as characters describing different situations (again depicted in speech bubbles), for example, "Mitsu says there is a hedgehog on the ground" and "Mary says there is a snake under the bed". Again, in instances of errors, the child was shown the correct response and congratulated when indicating it.

In the fifth activity, the objects described in activity 4 were often replaced by objects that they resembled, giving rise to characters being led astray. In these instances, false complements would be produced, e.g.: "Look, there is a brush on the ground, but Mitsu says that there is a hedgehog on the ground", or: "Look, there is a green sock under the bed, but Mary says that there is a snake under the bed". The characters' inaccurate descriptions of reality become plausible in this activity given the visual similarity of the objects. This exercise was a truth-value judgment task, with the child simply being asked to determine if the character was right or wrong. In instances when children made mistakes, a voice explained why this was so. Children were all encouraged to repeat the whole complement structure at the end of each exercise, such as "Here Mary says that there is a snake under the bed, but it is actually a sock" (Figure ii).

Supplementary Figure S2: Examples of activity 5, DIRE

Appendix B. Maximum scores and means (standard deviation) on tasks at pre-test and post-test in the syntactic training group for easier items (ToM precursors, true belief/complements)

	Maximum score	Pre-test	Post-test
ToM precursors	6	4.8 (1.5)	5.4 (1.2)
Verbal True Belief	6	5.5 (1.1)	5.3 (1.1)
Low- Verbal True Belief	6	5.1 (0.9)	5.6 (0.8)
True Complements	6	5.1 (1.2)	5.7 (1.2)

Appendix C: Detailed information about participants included

Population	Training	Age	Raven Score (SD)	Morphosyntax Score (SD)
TD	lexical	5.2	21 (0.7)	10 (-0.4)
TD	lexical	3.8	10*	11 (1)
TD	lexical	4.5	15 (0.2)	12 (1)
TD	lexical	4.8	14 (-0.1)	7 (-1.3)
TD	lexical	3.0	7*	9 (0.8)
TD	lexical	5.0	13 (-1)	10 (-0.4)
TD	lexical	5.3	17 (-0.1)	13 (0.6)
TD	lexical	4.6	25 (2.8)	14 (1.4)
TD	lexical	2.9	5*	10 (1.1)
TD	lexical	3.6	18*	12 (1.4)
TD	lexical	4.5	15 (0.2)	8 (-0.5)
TD	lexical	5.0	14 (-0.8)	11 (-0.1)
TD	syntax	4.0	14 (0.1)	10 (0.2)
TD	syntax	4.7	15 (0.2)	11 (0.2)
TD	syntax	5.1	17 (-0.1)	14 (0.9)
TD	syntax	4.9	14 (-0.8)	8 (-0.9)
TD	syntax	4.1	13 (-0.1)	11 (0.6)
TD	syntax	3.9	13 (-0.1)	7 (-0.4)
TD	syntax	4.8	16 (0.4)	14 (1.4)
TD	syntax	4.7	18 (1)	11 (0.2)
TD	syntax	4.3	13 (-0.3)	9 (-0.1)
TD	syntax	3.7	9*	10 (0.6)
TD	syntax	3.5	7*	5 (-0.6)
TD	syntax	4.0	6 (-2)	10 (0.2)
ASD	lexical	6.6	17 (-0.8)	-
ASD	lexical	10.0	18 (-3.1)	10**
ASD	lexical	11.5	19 (-3.4)	11**
ASD	lexical	9.8	17 (-3.3)	9**
ASD	lexical	6.1	8 (-2.9)	11**
ASD	lexical	9.9	21 (-2.4)	9**
ASD	lexical	7.5	9 (-2)	11**
ASD	lexical	10.9	16 (-4.6)	11**
ASD	syntax	9.5	8 (-5.4)	12**
ASD	syntax	7.3	13 (-1.5)	10**
ASD	syntax	6.2	15 (-1.4)	13**
ASD	syntax	7.0	23 (0)	15**
ASD	syntax	5.6	13 (-1)	7 (-2.4)
ASD	syntax	6.9	13 (-1.7)	9**
ASD	syntax	11.0	26 (-1.6)	8**
ASD	syntax	6.8	19 (-0.5)	-
SLI	lexical	6.3	13 (-1.6)	12**
SLI	lexical	8.2	15 (-2.8)	9**
SLI	lexical	5.3	19 (0.4)	9 (-0.8)
SLI	lexical	8.0	23 (-1)	11**
SLI	lexical	6.3	22 (0.1)	15**
SLI	lexical	7.2	10 (-2.2)	11**
SLI	lexical	7.0	18 (-0.9)	11**
SLI	lexical	8.5	20 (-1.5)	15**
SLI	lexical	6.8	17 (-0.8)	13**
SLI	lexical	9.0	20 (-2.6)	13**
SLI	syntax	6.1	11 (-2.3)	10**
SLI	syntax	4.9	13 (-1)	14 (1.4)
SLI	syntax	5.8	20 (0.6)	13 (0.3)
SLI	syntax	7.4	19 (-0.6)	13**
SLI	syntax	9.4	18 (-3)	12**
SLI	syntax	5.1	21 (0.7)	9 (-0.8)
SLI	syntax	5.2	17 (-0.1)	10 (-0.4)
SLI	syntax	4.8	17 (0.7)	9 (-0.5)
SLI	syntax	7.3	15 (-1.2)	9**
SLI	syntax	9.8	30 (-0.2)	15**

* no norms for this age (norms begin at age 3;9) ; ** no norms for this age (norms stop at age 6;0)

Appendix D: Individual results

Training	Group	Age of participants	Verbal FB			low-verbal FB			False complements		
			**Pre-test**	**Post-test**	**Follow-up**	**Pre-test**	**Post-test**	**Follow-up**	**Pre-test**	**Post-test**	**Follow-up**
			**/6**	**/6**	**/6**	**/6**	**/6**	**/6**	**/6**	**/6**	**/6**
**Syntactic training**	**TD group**	3.5	0	0		1	1		1	0	
		3.7	1	1		2	0		1	0	
		3.9	1	3	4	2	5	3	1	6	5
		4.0	0	1		4	0		0	3	
		4.0	0	5	6	6	5	5	0	6	5
		4.1	1	6	2	4	5	5	2	6	6
		4.3	0	6	6	2	5	5	0	6	6
		4.7	1	6	6	4	6	6	2	6	5
		4.7	0	6	6	5	6	4	0	6	6
		4.8	1	5	3	1	6	6	1	6	6
		4.9	0	6	6	0	6	5	3	5	4
		5.1	1	6	6	0	6	6	0	6	6
	**ASD group**	5.6	0	2	1	0	2	2	0	6	6
		6.2	0	6	6	3	6	6	2	6	6
		6.8	0	0		6	6		1	2	
		6.9	1	0	0	2	4	4	0	6	5
		7.0	2	5	5	1	0	0	1	6	5
		7.3	0	1	3	4	5	4	2	5	5
		9.5	3	2	5	4	3	3	2	3	4
		11.0	0	6	5	5	6	6	4	4	4
	**DLD group**	4.8	0	1		2	5		1	3	
		4.9	0	5		1	5	4	3	5	5
		5.1	1	3	1	5	6	6	3	6	6
		5.2	2	6	6	2	6	6	2	6	6
		5.8	0	6	1	0	3	5	0	5	5
		6.1	1	3	2	3	6	0	2	4	5
		7.3	1	0		3	3		0	5	
		7.4	1	1	0	0	5	5	0	6	5

9.4	1	0	0	1	6	2	4	6	6
9.8	0	5		5	6		6	6	

Training	Group	Age of participants	Verbal FB		Low-verbal FB		False complements	
			**Pre-test /6**	**Post-test /6**	**Pre-test /6**	**Post-test /6**	**Pre-test /6**	**Post-test /6**
**Lexical training**	**TD group**	2.9	0	1	3	2	3	2
		3.0	0	0	0	1	1	1
		3.6	1	0	1	0	1	3
		3.8	1	0	1	0	0	0
		4.5	0	0	4	4	3	5
		4.5	1	0	6	6	0	0
		4.6	0	3	1	1	1	1
		4.8	0	0	2	0	0	1
		5.0	2	0	1	4	1	1
		5.0	1	6	5	3	2	6
		5.2	1	0	1	0	0	0
		5.3	2	0	5	6	2	1
	**ASD group**	6.1	1	0	4	0	3	1
		6.6	0	0	5	5	0	0
		7.5	1	1	2	1	1	1
		9.8	4	4	4	3	3	3
		9.9	1	0	6	5	5	4
		10.0	3	0	4	4	3	5
		10.9	0	0	2	0	1	1
		11.5	2	1	6	6	1	1
	**DLD group**	5.3	0	0	6	5	3	2
		6.3	4	4	5	5	2	3
		6.3	0	0	0	0	0	0
		6.8	1	0	0	0	0	0
		7.0	0	0	5	6	3	6
		7.2	0	0	1	5	2	6

	8.0	0	0	0	1	0	3
	8.2	0	0	1	0	0	0
	8.5	0	0	1	0	5	4
	9.0	2	1	0	1	1	0
