# Supplementary figures and images for: The Impact of Grammar on Mentalizing: A Training Study Including Children With Autism Spectrum Disorder and Developmental Language Disorder
Source: Front Psychol. 2019 Nov 19;10:2478. doi: 10.3389/fpsyg.2019.02478 (PMC6877902; doi:10.3389/fpsyg.2019.02478)

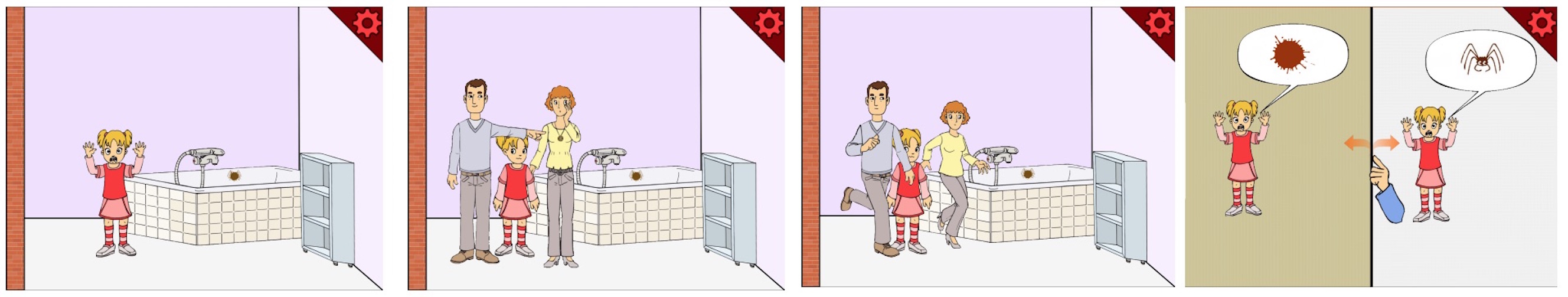

Supplement: Supplementary file 2 [file Image_1.jpeg]

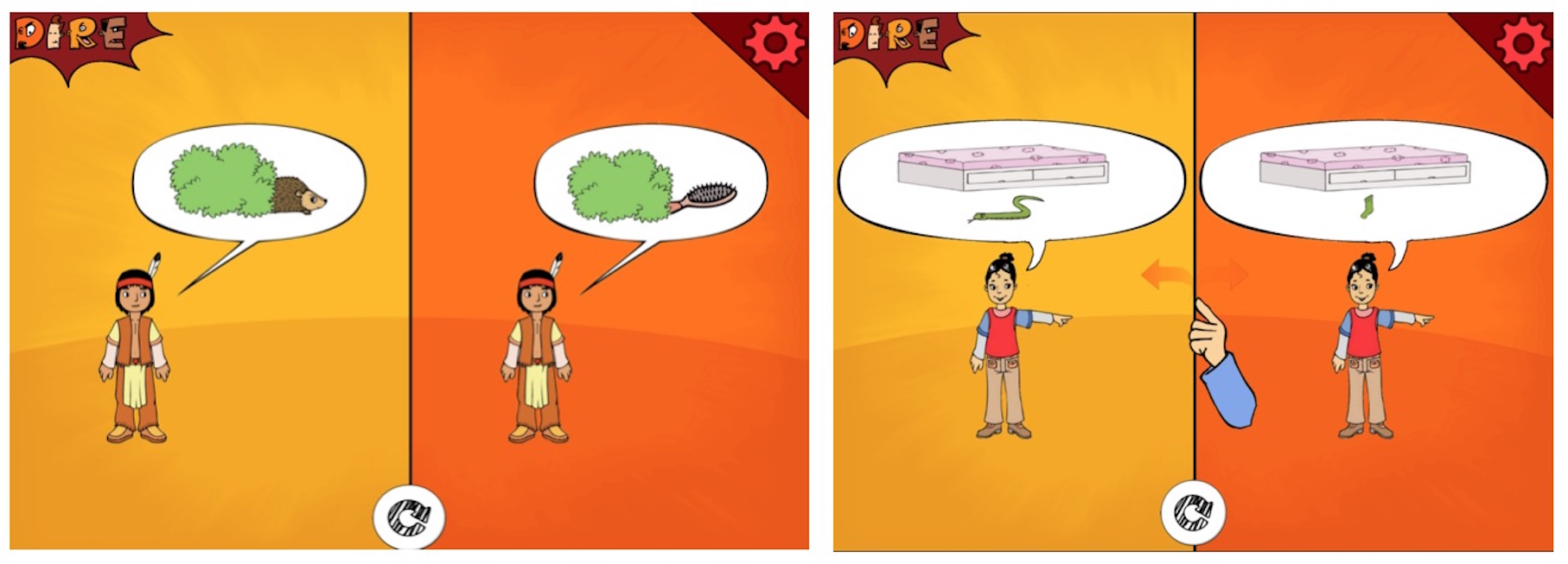

Supplement: Supplementary file 3 [file Image_2.jpeg]
